# The effects of age and ethnicity on the circadian variation of catecholamines and cortisol in employed women

**DOI:** 10.1186/s40695-018-0040-3

**Published:** 2018-06-28

**Authors:** Gary D. James

**Affiliations:** Department of Anthropology, Binghamton University-SUNY, Binghamton, NY 13902 USA

**Keywords:** Catecholamines, Cortisol, Aging, Ethnic variation, Women

## Abstract

**Background:**

Women employed outside the home in urban settings must adapt to changing circadian microenvironments. The pattern and extent of vasoactive hormone responses to these changes may depend upon age and ethnic background. The purpose of this study was to evaluate the effects of age and ethnicity on the circadian variation of urinary norepinephrine, epinephrine, and cortisol excretion across work, home and sleep microenvironments.

**Methods:**

The subjects of the study were 95 women (38 European-American, age = 35.4 ± 7.4; 28 African-American, age = 33.4 ± 7.9; 12 Asian-American, age = 36.7 ± 9.3 and 17 Hispanic-American age = 31.6 ± 10.9) employed as secretaries, lab technicians or office supervisors in New York City. Variation in the hormones across the microenvironments was evaluated using repeated measures ANCOVA with age group (18–29.9; 30–39.9; 40–49.9) and ethnicity as fixed factors.

**Results:**

The results show that for norepinephrine and epinephrine, work excretion rates are substantially higher than sleep rates (*p* < .001), and for epinephrine home rates were higher than sleep rates (*p* < .001). Work and sleep cortisol excretion rates were also significantly higher than the rate at home, consistent with cortisol’s circadian rhythm. (*p* < .002). Women in their twenties had substantially lower norepinephrine excretion rates than women over 30 (*p* < .04). There were also ethnic differences in norepinephrine (*p* < .04) and epinephrine (*p* = .11) output with Asian-American women having the lowest and African-American women having the highest rates. This variation is likely related to the ethnic variation in weight. There was no significant variation in cortisol excretion with age or ethnicity.

**Conclusion:**

The circadian rates of norepinephrine excretion differ by age and that of both catecholamines differ by ethnicity among women working outside the home. It is speculated that the age variation in norepinephrine may contribute to the development of vasomotor symptoms.

## Background

As women in Western industrialized society age, the qualitative nature of the social and physical environments to which they must adapt also changes [1]. For example, women’s social roles change as they age; they may begin their adult lives living single, then may marry and have children and as those children age, new challenges will emerge [1–3]. The question arises as to how women adapt to role change over the lifespan, or put another way how might the lifestyle changes that women experience into midlife affect their adaptive biology in ways that could affect their health in later life?

The sympathetic adrenal medullary system, as reflected in catecholamine secretion and the hypothalamus pituitary adrenal axis as reflected in cortisol secretion play major roles in regulating numerous bodily functions and are key in adapting people to their environments. The measureable products of the systems (hormones) are allostatic, meaning that circulating levels constantly change to meet external and internal environmental demands rather than maintaining a specific value (as in homeostasis). As people move through different microenvironments over a day, the levels of theses hormones will change to meet the social and physical demands. Over time, repeated over- and/or under- secretion of these hormones in response to constantly changing daily conditions can lead to allostatic load (a breakdown of regulated bodily functions) contributing to the development of chronic degenerative diseases [1]. Thus, understanding the circadian variation of these hormones in people of different ages and ethnicity can provide insight into not only how degenerative conditions such as hypertension, CVD, or diabetes develop, but also why they may develop differently in diverse groups [4].

There have been few studies examining the impact of aging on the real life circadian variation of biological functions that undergo allostasis in women working outside the home. James and Bovbjerg [2] reported that the effects daily perceived stress on circadian blood pressure variation did not differ among normotensive women employed outside of the home regardless of their age, however, they also showed that age had a U-shaped relationship with ambulatory blood pressures measured at work, home, and during sleep, such that women in their thirties had lower pressure that those in their twenties and forties [2]. They suggested that this age variation was due to the differential impact of childcare (different needs of children of different ages), as well as other factors such as the use of oral contraceptives particularly among younger women. Interestingly, the impact of a variety of social and behavioral stressors on the circadian variation of allostatic functions such as blood pressure, and catecholamine and cortisol excretion in women employed outside the home have been well documented, but these studies have not parsed out the effects in different age groups [1, 3, 5–8].

The circadian variation of allostatic hormones in women of different ethnicity have also been studied [9–11]. Ethnic comparisons, however, are often limited to just two groups, one of which is invariably European-American. Comparisons of other ethnic groups with each other, such as comparisons between Hispanic-American or Asian-American women with African-American women are rarely evaluated [11].

The purpose of this study was to evaluate the effects of age and ethnicity on the allostatic responses of urinary epinephrine, norepinephrine, and cortisol excretion across three changing daily microenvironments in women from four ethnic groups African-American, Asian-American, Hispanic-American and European-American, all of whom were employed outside the home in similar occupations at a single workplace.

## Methods

### Study design

Human biological studies that evaluate allostasis in everyday life often employ a “natural experimental” approach that features naturally occurring environmental contrasts [4, 12–14]. Many researchers have shown that the urban environment is heterogeneous, with various distinct microenvironments [4, 12, 15, 16]. There are often clear and stark boundaries between these microenvironments that define them as separate conditions, so that a “natural experiment” can be set up by contrasting biological responses across them [4, 12, 13]. Work (place of employment) and home (place of residence) have been firmly established as microenvironmental conditions that are useful in studying physiological or endocrine changes in everyday life [12, 13, 16–18]. In the work setting, social interactions mostly occur with non-related individuals, there is a specific occupational hierarchy that dictates social relationships, and there is a general conformity of behavior [4]. The conditions within this microenvironment contrast sharply with the home microenvironment, where domestic tasks and leisure activity occur in a context where social interactions are largely with neighbors and family members [4, 16]. The changes in physiological or endocrine measures across the work and home microenvironments can be evaluated as a “natural experiment” in which the response to the stressors associated with paid employment and domestic life are compared. A microenvironment that is similar for all subjects (such as overnight sleep, or more specifically, lying quietly in a dark room) can act as a pseudo-baseline in this “natural experiment” for biological responses, although with urinary cortisol, the evening home environment is more appropriate for this purpose, given the circadian rhythm of cortisol (see [17] for discussion).

For this study, the naturally occurring and easily definable microenvironmental variation inherent in the cosmopolitan New York City area was used to compare urinary catecholamine and cortisol excretion across work, home and sleep microenvironments. To add further control to the study, occupational location and type were also limited [4].

A limitation of the research design is that there is a consistent order of data collection such that data were collected at work first, followed by home and then sleep [4]. Studies have found that stress at work could carry over and elevate physiologic measures at home and during sleep [19]. But, if the sleep “baseline” before the work condition was used, possible carry-over effects of work the previous day on that sleep which would add an unknown bias to the comparisons.

To evaluate the impact of age and ethnicity on the microenvironmental variation of the urinary catecholamines and cortisol, the study subjects were cross classified into groups based on their age group (trichotomized by decade of life − 18-29.9; 30–39.9; and 40–49.9 years) and reported ethnicity.

### Subjects

The subjects of this study were 95 women (28 African-American, 12 Asian-American, 17 Hispanic-American and 38 European-American) employed as secretaries, laboratory technicians or office supervisors (all sedentary supervised occupations) at a major medical center in Manhattan in New York City who were participants in a larger protocol that was designed to assess the cardiovascular effects of life stress in women working outside the home. The women were studied between November and May each year from 1994 to 1998 and were examined on typical mid-week workdays (usually Tuesday thru Thursday). They were all volunteers that met several criteria in order to be eligible for study. Exclusion criteria in the larger protocol included: diagnosis of hypertension, cardiovascular disease, or diabetes, being pregnant or obese (defined as having > 40% of body mass as fat as determined from skinfold measurements), being on drug therapy (except oral contraceptives) or exhibiting significant premenstrual symptoms (defined by clinical treatment for them) [20]. Women using oral contraceptives were excluded from this study. The subjects ranged in age from 18 to 50 years. The study was approved by the IRB at the Weill College of Medicine of Cornell University and all subjects provided informed consent. Table 1 shows selected biological and demographic characteristics of the study sample.

**Table 1:** Selected Characteristics^a^ of the Study Sample (*N* = 95)

Characteristic	African American	Asian American	Hispanic American	European American
N	28	12	17	38
Age (years)	33.4 ± 7.9	36.7 ± 9.3	31.6 ± 10.6	35.4 ± 7.4
Height (Cms.)	163.9 ± 5.5	159.7 ± 6.3	159.2 ± 5.8	164.9 ± 7.8
Weight (Kgs.)	72.2 ± 15.0	60.3 ± 12.4	64.1 ± 10.1	65.4 ± 10.3
Triceps Sk. (Mm.)	24.9 ± 6.1	21.8 ± 6.7	23.1 ± 7.3	21.9 ± 6.6
Subscapular Sk. (Mm.)	24.2 + 11.6	22.6 + 9.8	21.8 + 7.8	19.1 + 7.1
Education (years)	14.6 + 1.5	17.1 + 1.7	15.4 + 2.4	16.1 + 2.5
Married (%)	28.6	66.7	29.4	36.8
Smokers (%)	17.9	0.0	17.6	21.1

^a^Mean ± standard deviation or percentage

As indicated, the African-American women are on average taller and weigh more than the other groups, while the Asian-American women are the shortest and weigh the least. The Asian-American women also differ from the other three groups in that a greater proportion of them are married and a much smaller proportion of them smoke.

### Protocol

The procedures used to collect the data has been detailed elsewhere [20]. In brief, subjects arrived at the Hypertension Center of New York Hospital at the beginning of their workday (between 8 and 9 AM) where height, weight and a series of anthropometric measurements were taken and demographic data, medical history, life stress, and psychometric information was also collected. At about 11 AM, the women were contacted at their work place and asked to empty their bladder, but to not collect that specimen. The time of this urination was recorded, signifying the beginning of the work period. The subjects were then given a 3-l polyethylene bottle and instructed to collect all their urine for the next 4 h. At 3 PM the subjects were again contacted and asked to empty their bladder, but into the polyethylene bottle. The time of this collection was documented as the end of the work period. At this time the women were given two additional 3-l polyethylene bottles for collecting urine at home in the evening and overnight (sleep). They were instructed to empty their bladder upon arriving at home (not collected) and to note the time. They were then to collect all their urine until bedtime (at approximately 10 PM). The time of this collection was noted and defined the end of the home period and beginning of the sleep period. The subjects were instructed to empty their bladder into the remaining polyethylene bottle upon awakening (at approximately 6 AM), noting the time. This time represented the end of the sleep period. These home and sleep samples were then returned that morning to the Hypertension center. Each subject thus had three environmentally tethered samples: at work, home and during sleep.

The urine sample bottles contained.5 g of sodium metabisulphite (a preservative for the catecholamines). This preservative has been widely used in field studies of urinary catecholamine variation [4, 10, 21, 22]. Methods used to assay the catecholamines are detailed elsewhere [21] and the rates of catecholamine excretion are expressed as nanograms/minute. The concentration of cortisol was determined using a solid phase ^125^I radioimmunoassay [23] and was expressed as μg/24 h. The preservative used for the catecholamines has no known effect on the cortisol assay [24].

### Analysis

To evaluate the effects of age and ethnicity on the circadian variation in urinary norepinephrine, epinephrine, and cortisol across the work, home and sleep microenvironments, a repeated measures ANCOVA was employed with microenvironment as a repeating factor, and age group (18–29.9; 30–39.9 40–49.9) and ethnicity as fixed factors. Because the sizes of the three-way interaction groups are very small, the models only included and tested main effects and two-way interactions that included microenvironment. A single body fat measure (subscapular skinfolds) was included as a covariate in the models. Statistical significance was set at *p* < .05. Post-hoc group comparisons were adjusted for multiple comparisons using the Bonferroni method. All analysis was conducted using SPSS 19.

## Results

Tables 2, 3, and 4 show the mean rates of urinary norepinephrine, epinephrine and cortisol excretion at work, home, and sleep among the four ethnic groups by age group. Among all women and within each age and ethnic group, urinary norepinephrine and epinephrine excretion at work are significantly greater than during sleep (*p* < .001) and for epinephrine, home excretion is also significantly greater than sleep (*p* < .001). In addition, overall and within each age and ethnic group, the rates of Cortisol excretion at work (*p* < .001) and during sleep (*p* < .002) are significantly greater that the rate of excretion at home.

**Table 2:** Comparisons of Norepinephrine Excretion Rate (ng/min) at Work, Home, and during Sleep by Age Group and Ethnicity^a^

Ethnicity											Age Group										
	18–29.9							30–39.9							40–49.9						
	Work			Home		Sleep		Work			Home		Sleep		Work			Home		Sleep	
	N	$\overline{\mathrm{x}}$	SD	$\overline{\mathrm{x}}$	SD	$\overline{\mathrm{x}}$	SD	N	$\overline{\mathrm{x}}$	SD	$\overline{\mathrm{x}}$	SD	$\overline{\mathrm{x}}$	SD	N	$\overline{\mathrm{x}}$	SD	$\overline{\mathrm{x}}$	SD	$\overline{\mathrm{x}}$	SD
European-American	7	17.22	10.26	17.81	14.46	6.24	1.51	18	38.04	46.85	31.51	34.45	16.84	12.56	13	25.89	11.37	21.35	11.30	14.31	7.36
African-American	12	26.47	14.24	23.64	14.14	12.56	15.15	9	22.31	11.87	25.39	21.46	13.65	6.25	7	35.35	22.52	40.57	31.67	13.10	4.79
Asian-American	3	8.90	3.13	8.80	5.05	3.40	1.25	4	29.78	5.09	21.10	10.75	10.04	1.76	5	13.50	1.77	14.85	2.92	7.33	1.65
Hispanic American	8	27.06	15.32	14.45	10.09	6.59	3.50	5	16.63	3.49	15.85	7.52	5.25	1.61	4	29.67	15.77	23.16	13.84	11.90	7.49
Total	30	22.71	13.93	18.35	13.07	8.58	10.11	36	30.21	34.29	26.65	27.02	13.68	10.15	29	26.56	15.57	25.12	19.42	12.48	6.40

$\overline{\mathrm{x}}$ mean, *SD* standard deviation

^a^Work, Home>Sleep by ethnicity, age and overall, *p* < .001

**Table 3:** Comparisons of Epinephrine Excretion Rate (ng/min) at Work, Home, and during Sleep by Age Group and Ethnicity^a^

Ethnicity											Age Group										
	18–29.9							30–39.9							40–49.9						
	Work			Home		Sleep		Work			Home		Sleep		Work			Home		Sleep	
	N	$\overline{\mathrm{x}}$	SD	$\overline{\mathrm{x}}$	SD	$\overline{\mathrm{x}}$	SD	N	$\overline{\mathrm{x}}$	SD	$\overline{\mathrm{x}}$	SD	$\overline{\mathrm{x}}$	SD	N	$\overline{\mathrm{x}}$	SD	$\overline{\mathrm{x}}$	SD	$\overline{\mathrm{x}}$	SD
European-American	7	5.08	7.08	2.92	2.09	0.88	0.50	18	6.25	4.57	4.93	3.78	3.74	6.55	13	5.03	3.42	3.55	2.43	4.62	5.34
African-American	13	6.33	3.44	4.84	3.89	3.30	3.57	9	4.91	1.85	5.21	5.59	1.57	1.08	6	6.72	7.58	3.39	2.36	1.82	2.16
Asian-American	3	3.02	1.65	1.73	1.51	0.39	0.33	4	6.21	2.52	3.03	2.04	1.15	0.88	5	2.94	1.56	1.81	2.40	0.59	0.29
Hispanic American	8	8.13	2.38	4.17	3.01	1.34	1.03	5	3.49	2.02	2.41	2.57	0.47	0.32	4	5.36	1.60	3.49	2.48	1.09	0.60
Total	31	6.19	4.81	3.93_	3.20	1.96	2.61	36	5.53	3.60	4.44_	4.03	2.46	4.80	28	5.07	4.24	3.20	2.37	2.80	4.09

$\overline{\mathrm{x}}$ mean, *SD* standard deviation

^a^Work, Home>Sleep by ethnicity, age and overall, *p* < .001

**Table 4:** Comparisons of Cortisol Excretion Rate (μg/24h) at Work, Home, and during Sleep by Age Group and Ethnicity^a^

Ethnicity											Age Group										
	18–29.9							30–39.9							40–49.9						
	Work			Home		Sleep		Work			Home		Sleep		Work			Home		Sleep	
	N	$\overline{\mathrm{x}}$	SD	$\overline{\mathrm{x}}$	SD	$\overline{\mathrm{x}}$	SD	N	$\overline{\mathrm{x}}$	SD	$\overline{\mathrm{x}}$	SD	$\overline{\mathrm{x}}$	SD	N	$\overline{\mathrm{x}}$	SD	$\overline{\mathrm{x}}$	SD	$\overline{\mathrm{x}}$	SD
European-American	7	45.22	40.82	26.14	33.00	13.12	6.05	17	41.97	30.51	30.55	39.60	29.84	14.58	12	36.91	23.82	21.89	39.60	33.48	13.73
African-American	13	43.12	28.12	18.13	13.02	47.09	60.76	8	26.04	21.34	26.85	32.59	37.00	36.82	7	52.0	64.46	23.04	24.69	16.60	5.97
Asian-American	3	19.21	9.26	8.71	3.33	25.93	18.14	4	82.14	61.76	15.52	5.22	54.22	28.09	5	37.05	26.42	16.96	12.47	59.49	47.82
Hispanic American	8	66.86	49.38	22.69	16.96	26.07	17.52	5	24.13	15.40	8.58	5.81	21.05	6.50	4	27.13	7.08	27.21	25.26	36.91	32.35
Total	31	47.41	37.60	20.20	19.45	31.95	42.10	34	40.33	35.01	24.68	32.57	33.10	23.51	28	39.32	36.48	22.06	20.39	34.39	27.20

$\overline{\mathrm{x}}$ mean, *SD* standard deviation

^a^Work>Home Sleep by ethnicity, age and overall, *p* < .001; Sleep>Home bu ethnicity, age and overall, *p* < 002

**Fig. 1:**
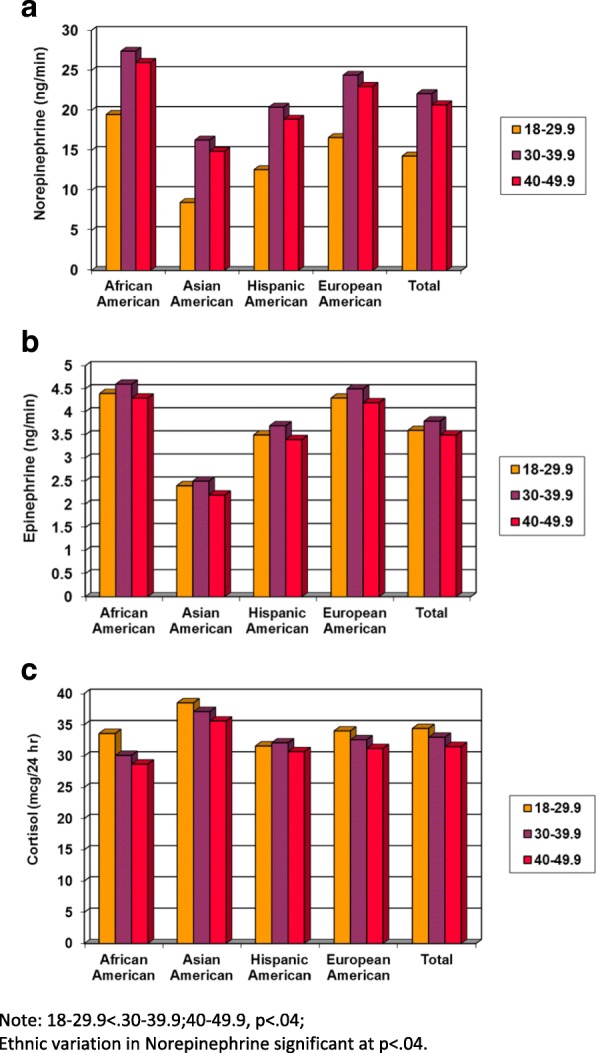
Variation in the Urinary Rates of Norepinephrine (ng/min), Epinephrine (ng/min), and Cortisol (μg/24 h) Excretion by Age Group (18-29.9, 30-39.9, 40-49.9 years) and Ethnicity

The main effects of age and ethnicity on the rates of urinary catecholamine and cortisol excretion averaged over the whole day are illustrated in Fig. 1a-c. As indicated, women in the youngest age group (18–29.9 years) had significantly lower norepinephrine excretion rates that than women in the two older age groups, regardless of ethnic background (*p* < .04). There were also significant (*p* < .04) ethnic differences in average norepinephrine output, with Asian-American women having substantially lower rates than the other groups, and African-American women tending to have the highest. The daily average rates of epinephrine excretion had a similar but not statistically significant circadian pattern by ethnic group as norepinephrine excretion (*p* = .11) and there was no statistically significant age effect (*p* = .91). There was no statistically significant variation in cortisol with age (*p* = .87) or ethnicity (*p* = .89).

## Discussion

The results show that the patterns of urinary catecholamine and cortisol excretion across the daily microenvironments are similar by age group and ethnicity, indicating that the allostatic, physiological response to the stressors associated with microenvironmental changes during the day is similar by age and ethnicity. These results confirm to some extent of those of James and Bovbjerg [2], who found that perceived stress during the day had similar effects on microenvironmental blood pressure variation by age group.

However, of particular note is the stark differences in norepinephrine excretion between women in the youngest age group (18–29.9) and the two older age groups (women over 30 years of age), such that norepinephrine excretion is dramatically increased among women over 30 years of age. Many previous studies have found that there is an increase in plasma norepinephrine levels with age, which occurs for a variety of physiological reasons, but the studies on which the effect is based generally compare subjects in their twenties with those in their sixties/seventies [25]. What is interesting is the consistent nearly doubling of norepinephrine excretion from the twenties to thirties among all the ethnic groups.

Perhaps the increase in norepinephrine excretion at this time of life is related to the potential development of vasomotor symptoms through the menopausal transition, as an increase in brain norepinephrine plays a key role in the narrowing of the thermoneutral zone in midlife women [26]. The loss of estrogen also plays a role but is insufficient in and of itself to cause of vasomotor symptoms (such as hot flashes) [26]. One might speculate that the current data suggests that there is first an increase in norepinephrine which alone is insufficient to initiate vasomotor responses, but once estrogen begins its decline and gets to a certain level, the norepinephrine effects on thermoregulation are triggered. Further research is need to explore this possibility.

There was also significant ethnic variation in the total amount of norepinephrine and to some extent epinephrine excretion across the day, with the African American women excreting the highest amounts, with the Asian-American women excreting the least. The European-American and Hispanic American women had similar rates of excretion that were between the other two groups. While, it is possible that this group difference may reflect an overall difference in experienced stress, it is most likely that the differences are related to differences in body size and weight among the groups. Many studies have found a positive association between body mass and catecholamine excretion [25, 27]. The catecholamine differences in this study parallel the weight differences among the groups, and when weight is added to the model, ethnicity is no longer a statistically significant contributor to norepinephrine variance (*p* < 04 to *p* = .19 with weight in the model) and the effect for epinephrine is similar (*p* = .11 to *p* = .19). This suggests that the ethnic difference is related to the weight difference. Other epidemiological studies evaluating ethnic differences in 12 and 24-h urinary catecholamine measurements among both men and women have found no ethnic differences among European-American, African-American and Hispanic-American groups after adjusting for body size/muscle mass [28, 29].

Finally, the current study found no differences by age or ethnicity in the total amount of cortisol excretion over the day, nor in the patterns of urinary excretion across the daily microenvironments. This finding suggests that stressor related changes in cortisol across the microenvironments are similar by age and ethnicity. Epidemiological studies of salivary cortisol suggest that African-Americans and Hispanic-Americans have flatter diurnal profiles than European-Americans [30–32]; which has been largely attributed to socioeconomic differences. In this study, socioeconomic status (SES) is similar among the participants (all have similar educational backgrounds and all work in a limited number of supervised sedentary occupations at the same workplace). Thus, it is unlikely that SES is a significant contributor to the current results due to the narrow range in the sample. However, salivary and urinary measures also evaluate very different aspect of cortisol dynamics. Sequential salivary samples are point measures designed to describe a circadian rhythmic pattern, where urinary measures are integrated over both a timeframe and an environmental circumstance and are designed to reflect the average levels in those contexts [18]. This difference in the perspective of the measurements may make them not comparable.

Caution should be used in extrapolating the results of this study to the general population, as the sample was limited to healthy younger women employed in select occupations. In addition, ethnicity was determined by self-report, which could increase type II error in the group comparisons. The sample sizes of the groups, particularly by ethnicity were also small, thus affecting the ability to detect significant differences among the groups. Nonetheless, the findings show that daily hormonal responses to changing daily microenvironments as reflected in urinary excretion rates are similar by ethnicity and by age in premenopausal women working outside the home. The results also show that there is a dramatic increase in norepinephrine excretion among women in their thirties, which may presage vasomotor symptoms as women transition through menopause. There are also significant differences in the level but not circadian pattern of catecholamine excretion rates by ethnicity, which may reflect differences in average body size among the groups. Further research is needed to verify these findings.

## Conclusion

The circadian rates of norepinephrine excretion differ by age and that of both catecholamines may differ by ethnicity among women working outside the home. It is speculated that the age variation in norepinephrine may contribute to the development of vasomotor symptoms.
